# Bidirectional Promoter-Based CRISPR-Cas9 Systems for Plant Genome Editing

**DOI:** 10.3389/fpls.2019.01173

**Published:** 2019-09-20

**Authors:** Qiurong Ren, Zhaohui Zhong, Yan Wang, Qi You, Qian Li, Mingzhu Yuan, Yao He, Caiyan Qi, Xu Tang, Xuelian Zheng, Tao Zhang, Yiping Qi, Yong Zhang

**Affiliations:** ^1^Department of Biotechnology, School of Life Sciences and Technology, Center for Informational Biology, University of Electronic Science and Technology of China, Chengdu, China; ^2^Jiangsu Key Laboratory of Crop Genetics and Physiology, Jiangsu Co-Innovation Center for Modern Production Technology of Grain Crops, Jiangsu Key Laboratory of Crop Genomics and Molecular Breeding, Agricultural College of Yangzhou University, Yangzhou, China; ^3^Key Laboratory of Plant Functional Genomics of the Ministry of Education, Joint International Research Laboratory of Agriculture and Agri-Product Safety of the Ministry of Education, Yangzhou University, Yangzhou, China; ^4^Department of Plant Science and Landscape Architecture, University of Maryland, College Park, MD, United States; ^5^Institute for Bioscience and Biotechnology Research, University of Maryland, Rockville, MD, United States

**Keywords:** CRISPR-Cas9, plant genome editing, rice, bidirectional promoter, enhancer

## Abstract

CRISPR-Cas systems can be expressed in multiple ways, with different capabilities regarding tissue-specific expression, efficiency, and expression levels. Thus far, three expression strategies have been demonstrated in plants: mixed dual promoter systems, dual Pol II promoter systems, and single transcript unit (STU) systems. We explored a fourth strategy to express CRISPR-Cas9 in the model and crop plant, rice, where a bidirectional promoter (BiP) is used to express Cas9 and single guide RNA (sgRNA) in opposite directions. We first tested an engineered BiP system based on double-mini 35S promoter and an *Arabidopsis* enhancer, which resulted in 20.7% and 52.9% genome editing efficiencies at two target sites in T0 stable transgenic rice plants. We further improved the BiP system drastically by using a rice endogenous BiP, OsBiP1. The endogenous BiP expression system had higher expression strength and led to 75.9–93.3% genome editing efficiencies in rice T0 generation, when the sgRNAs were processed by either tRNA or Csy4. We provided a proof-of-concept study of applying BiP systems for expressing two-component CRISPR-Cas9 genome editing reagents in rice. Our work could promote future research and adoption of BiP systems for CRISPR-Cas-based genome engineering in plants.

## Introduction

CRISPR-Cas9 and Cas12a (formerly Cpf1) are widely used sequence-specific nucleases (SSNs) for plant genome editing (Jiang et al., 2013; Li et al., 2013; Nekrasov et al., 2013; Fauser et al., 2014; Endo et al., 2016; Xu et al., 2016; Begemann et al., 2017; Tang et al., 2017; Zhou et al., 2017; Lowder et al., 2018; Tang et al., 2018; Zhong et al., 2018; Zhong et al., 2019; Zhou et al., 2019). Unlike meganucleases, zinc finger nucleases (ZFNs), and TAL effector nucleases (TALENs), a CRISPR-Cas system relies on a single guide RNA (sgRNA, for Cas9) or CRISPR RNA (crRNA, for Cas12a) for DNA targeting, bypassing protein engineering. This easiness has made CRISPR-Cas systems as top SSN choices for plant reverse genetics and accelerated crop breeding. While it is possible to deliver the Cas protein and the guide RNA as preformed ribonucleoprotein (RNP) complexes (Woo et al., 2015; Svitashev et al., 2016; Liang et al., 2017; Andersson et al., 2018), plant applications have largely relied on *Agrobacterium* mediated T-DNA transformation where Cas9/Cas12a and guide RNA expression cassettes are packaged into a T-DNA vector. Conventionally, a Cas gene is expressed by a Pol II promoter and a guide RNA is expressed by a Pol III promoter, and this system is termed as a mixed dual promoter system. However, Pol III promoters, such as U6 or U3, cannot match the expression strength of some strong Pol II promoters, which limits the overall genome editing efficiency in plants (Tang et al., 2016; Cermak et al., 2017; Mikami et al., 2017; Tang et al., 2019). Further, Pol III promoters are only suitable for expression of relatively short transcripts, which prevents the use of a single Pol III promoter to effectively express multiple sgRNAs for multiplexed genome editing, an important feature and advantage of CRISPR-Cas technologies.

To utilize Pol II promoters for guide RNA expression, two novel CRISPR-Cas expression systems have been developed in recent years. The first system, a dual Pol II promoter system, utilizes two separate Pol II promoter-terminator cassettes to express the Cas gene and the guide RNA. This system has been demonstrated for efficient plant genome editing with Cas9 (Cermak et al., 2017) and Cas12a (Tang et al., 2017; Zhong et al., 2018). However, repeated use of the same promoter may have a risk of gene silencing. Using two different Pol II promoters for Cas and guide RNAs may address this potential problem. Use of two separate promoters, however, adds to the length of the final DNA expression constructs. The second system, single transcript unit (STU), utilizes only one promoter to express the Cas gene and the guide RNAs for plant genome editing, as demonstrated for CRISPR-Cas9 (Tang et al., 2016; Mikami et al., 2017; Zhou et al., 2017; Tang et al., 2019) and CRISPR-Cas12a (Zhong et al., 2018; Tang et al., 2019). While STU is very compact, the expression system may not be optimal for the CRISPR-Cas system. This is because the same amount of Cas-sgRNA STU mRNAs is transcribed, yet it takes another protein translation step to generate Cas proteins. It hence may not be possible to achieve 1:1 molar ratio of Cas protein and guide RNA *in vivo* based on a STU expression system.

It would be useful to develop bidirectional promoter (BiP) systems for CRISPR-Cas expression. In this case, it only requires one promoter to express both Cas and guide RNAs (just as in a STU system). However, expression of either component can be independently fine-tuned with the use of different 3′-UTR or/and terminators (as with a dual Pol II system). Recently, a BiP CRISPR-Cas9 system, coupled with ribozyme-based sgRNA processing, was successfully developed for efficient genome editing in *Oleaginous Microalga* (Poliner et al., 2018). In this study, we explored BiP strategies for the expression of CRISPR-Cas9 for genome editing and sought to prove the concept in rice, which is a model plant and a major crop. We tested an engineered BiP system as well as a plant endogenous BiP system. Our study suggests promising applications of BiP systems for efficient expression of CRISPR-Cas systems in plant genome editing.

## Materials and Methods

### Vector Construction

The BiP CRISPR-Cas9 system plasmids were constructed using pTX152 (p35S::Hyg::35S T + p35S::Cas9::Hsp T) (Tang et al., 2016; Zhou et al., 2017) as a vector backbone. To construct Cas9-tRNA intermediate cloning vector (pGEL038), tRNA::ccdB::sgRNA::tRNA and Cas9 fragment were amplified from pGEL031 (Tang et al., 2019), and Ocs terminator was amplified from pZHY933 (Zhou et al., 2019). Next, these three fragments were cloned in between the *Asc*I and *Sbf*I sites of pTX152 (Tang et al., 2016; Zhou et al., 2017) by Gibson assembly. To construct Cas9-Csy4 intermediate cloning vector (pGEL039), Csy4 cleavage site::ccdb::gRNA scaffold::Csy4 cleavage site fragment and the Csy4-P2A fragment were amplified from pGEL031 (Tang et al., 2019) and then cloned in between the *Kpn*I and *Sbf*I sites of the Cas9-tRNA plasmid by Gibson assembly. To construct the mini 35s-Cas9-Csy4 system (pGEL050), two CaMV 35S minimal promoters were synthesized and further cloned into the *Spe*I and *Sbf*I sites of Cas9-Csy4 system by T4 ligase. To create the mini 35s-enhancer-Cas9-Csy4 system (pGEL051), the *Arabidopsis* enhancer (additional data: **Figure S4**) (Zhu et al., 2015; Zhang et al., 2016) was amplified from *Arabidopsis* Col-0, cloned into *Xba*I and *Pvu*II-linearized pGEL050 plasmids by T4 ligase. To generate the OsBiP1-Cas9-tRNA system (pGEL052) and OsBiP1-Cas9-Csy4 system (pGEL053), the OsBiP1 promoter fragment (additional data: **Figure S5**) (Wang et al., 2016) was amplified from *Oryza sativa* Geng/Japonica cultivar Nipponbare and cloned into pGEL038 and pGEL039 by Gibson assembly. For creating nuclease expression vector, sgRNAs were synthesized as duplexed oligonucleotides (**Table S1**). Oligos were annealed into *Bsa*I-linearized Cas9 vectors by Golden Gate cloning. The fragments of mini 35s–green-fluorescent protein (GFP) (pGEL056), mini 35s-enhancer-GFP (pGEL057), and OsBiP1-GFP (pGEL058) were respectively amplified from pGEL050, pGEL051, and pGEL052 and cloned into ZmUbi-GFP (pGEL055) by Gibson assembly. The vectors generated in this study are available at Yong Zhang Lab upon request.

### Rice Protoplast Transformation

The *O. sativa* Geng/Japonica cultivar Nipponbare was used in this study. Rice protoplast isolation and transformation with T-DNA vectors were performed according to our previously published protocols (Shan et al., 2013; Zhang et al., 2013; Zheng et al., 2016). Rice seedlings were grown at 28°C in the dark for 12 days. Thirty to 40 fresh rice seedlings were cut into 0.5- to 1-m strips with a razor blade and quickly transferred into 8–10 ml of enzyme solution (1.5% Cellulase R10, 0.75% Macerozyme R10, 0.6 M of mannitol, 10 mM of MES, 10 mM of CaCl_2_, and 0.1% bovine serum albumin (BSA), at pH 5.8). Vacuum infiltration was applied for 30 min and, then strips were digested by shaking at 60–80 rpm for 6–8 h at 25°C in the dark. The digested products were filtered with 40-μm nylon mesh into a 50-ml tube with 10 ml of W5 buffer. The protoplasts were collected through centrifugation at 100×*g* for 5 min and then resuspended with 10 ml of W5 buffer. This step was repeated with centrifugation at 100×*g* for 2 min at room temperature. The cells were then suspended in MMG buffer (0.4 M of mannitol, 4 mM of MES, and 15 mM of MgCl_2_, at pH 5.8) for 2.6 × 10^−6^ cells/ml. Two hundred microliters of protoplasts was mixed with 30 μl of plasmid (30 μg) and 230 μl of PEG buffer (40% w/v PEG4000, 0.2 M of mannitol, and 0.1 M of CaCl_2_) for an incubation of 20 min at room temperature. After 900 μl of W5 buffer to stop transformation was added, the protoplasts were centrifuged at 250×*g* for 5 min and resuspended in 1 ml of washing and incubation (WI) buffer (0.5 M of mannitol, 20 mM of KCl, and 4 mM of MES at pH 5.7), before being transferred into three 6-well culture plate for 32°C incubation. After incubation for 48 h, the protoplasts were collected for DNA extraction and further analysis. Each protoplast transformation experiment was performed in three biological replicates.

### Rice Stable Transformation

Rice stable transformation was conducted as published previously with the *Agrobacterium tumefaciens* strain EHA105 (Zheng et al., 2016; Zhou et al., 2017; Zhou et al., 2019). The rice seeds were sterilized with 70% ethanol for 1 min and washed with sterile water, and then 2.5% sodium hypochlorite containing a drop of Tween 20 was added for 15 min of shaking. After being washed with sterile water, these seeds were sterilized in 2.5% sodium hypochlorite for another 15 min. Afterwards, these seeds were washed with sterile water and cultured on solid medium at 28°C for 2–3 weeks. *Agrobacterium* cultures were resuspended in liquid medium (OD 600 = 0.06–0.1) containing 100 µM of acetosyringone. The fresh rice calli were chosen and immersed in the *Agrobacterium* liquid medium for 2 min. These calli were collected and cultured in solid medium at 25°C in a dark growth chamber. After 3 days, these calli were washed with sterile water and transferred onto screening medium at 32°C with a 12-h light/12-h dark photoperiod for 2 weeks. Then, growing calli were moved onto regenerative medium at 28°C with a 16-h light/8-h dark cycle. After 3–4 weeks, the transgenic rice plants were grown into seedlings for subsequent analyses.

### Mutation Analysis by CAPS

Genomic DNA was extracted from transformed rice protoplasts or transgenic rice using the cetyl trimethylammonium bromide (CTAB) method (Stewart and Via, 1993). Mutagenesis at target sites was analyzed by cleaved amplified polymorphic sequence (CAPS) with corresponding enzymes. The *OsPDS*-sgRNA01 site was amplified with primers *OsPDS*-F1 and *OsPDS*-R1, and the polymerase chain reaction (PCR) product was digested with the enzyme *Pst*I (**Table S1**). The *OsDEP1*-sgRNA01 site was amplified with primers *OsDEP1*-F and *OsDEP1*-R (**Table S1**) and then digested with the enzyme *Mfe*I. All digested products were resolved on 1% agarose gels.

### High-Throughput Sequencing Analysis

High-throughput sequencing analysis was carried out as published previously (You et al., 2018). Genome regions of targeted sites were PCR amplified using high-throughput primers (**Table S1**). The *OsPDS*-sgRNA01 site was amplified with primers Cas9-*OsPDS*-HTS-F1 and Cas9-*OsPDS*-HTS-R1. The *OsDEP1*-sgRNA01 site was amplified with primers Cas9-*OsDEP1*-HTS-F1 and Cas9-*OsDEP1*-HTS-R1. The PCR products were purified by Qubit 2.0 Fluorometer (Life Technologies) and then sequenced using Illumina HiSeq 2500 platform. Each sample generated more than 50,000 reads. Raw sequencing data were analyzed by CRISPRMatch (You et al., 2018). The mean averages and standard deviations of three biologically independent replicates were calculated. To compare two conditions, the two-tailed test was used, assuming unequal variance between samples.

## Results

### Genome Editing in Rice Cells With an Engineered BiP CRISPR-Cas9 System

**Figure 1:**
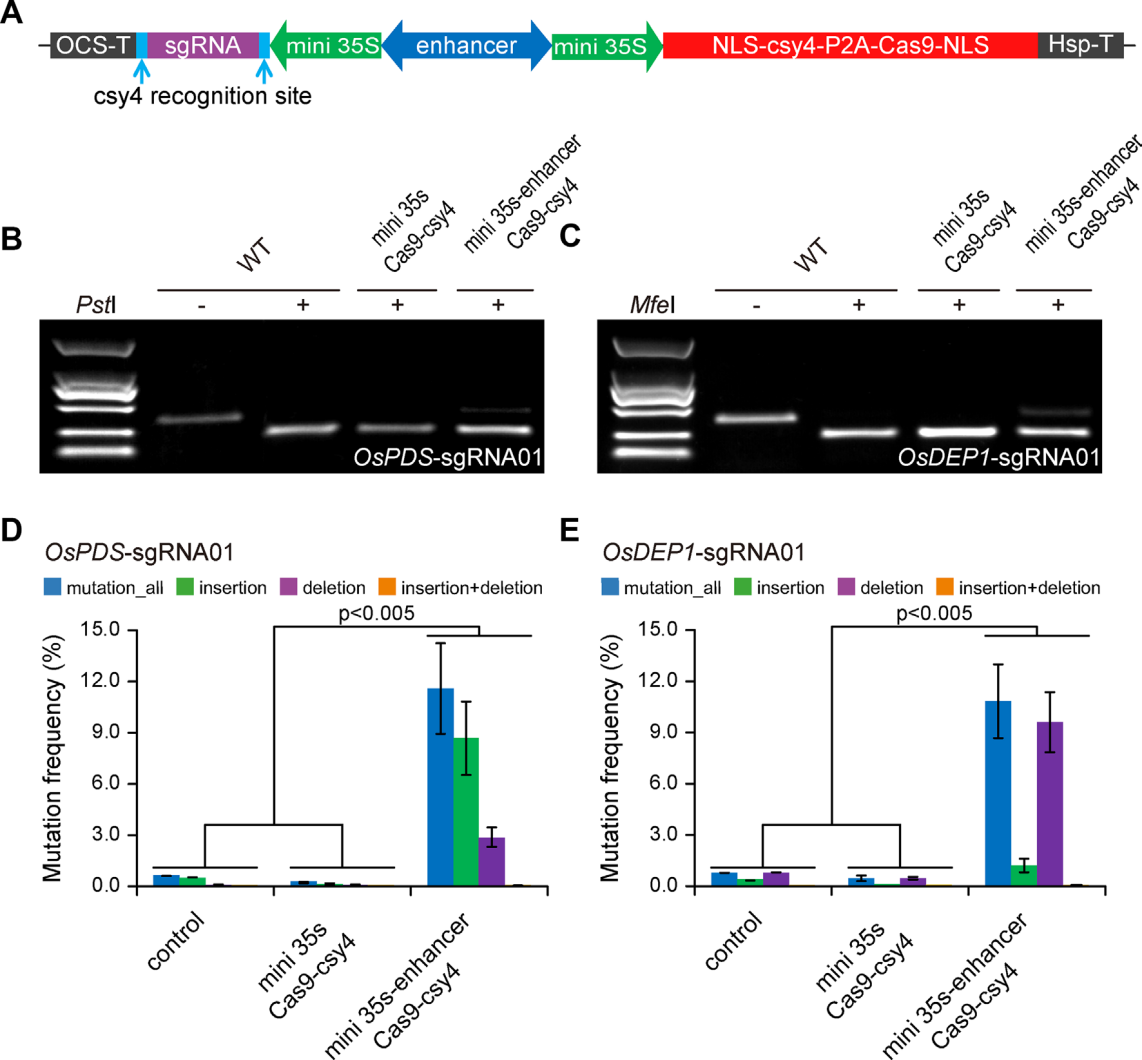
Expression of CRISPR-Cas9 with an engineered bidirectional promoter system **(A)** Diagrams of expression cassettes. **(B)** Cleaved amplified polymorphic sequence (CAPS) analysis of mutation frequencies at the *OsPDS* target site. **(C)** CAPS analysis of mutation frequencies at the *OsDEP1* target site. **(D)** Quantification of mutagenesis at the *OsPDS* target site by deep sequencing. **(E)** Quantification of mutagenesis at the *OsDEP1* target site by deep sequencing. Bar graphs show average mutation frequency from three biologically independent replicates with error bars representing standard deviations (*n* = 3). The statistical analyses were performed using the two-tailed test.

To demonstrate that we can expression CRISPR-Cas9 with a BiP, we first engineered a BiP based on CaMV 35s minimal promoter (Xie et al., 2001). As illustrated in **Figure 1A**, two CaMV 35s minimal promoters flanking an *Arabidopsis* enhancer sequence (Zhu et al., 2015; Zhang et al., 2016) were positioned in opposite directions, with one driving Cas9 expression and the other one driving the sgRNA. As a negative control, a minimal BiP without the *Arabidopsis* enhancer sequence was used. A BiP, unlike Pol III promoters such as U6 or U3, utilized RNA polymerase II for transcription. We used a Csy4 RNase system for precise processing of the sgRNA (Tsai et al., 2014; Cermak et al., 2017), so that it will not have a 5′ cap and 3′ poly A sequence. We designed one sgRNA each for targeting *OsPDS* and *OsDEP1* in rice. The resulting T-DNA constructs were used to transform rice protoplasts. Targeted mutagenesis was assessed by CAPS analysis, where uncut bands indicate targeted mutations induced by Cas9. The results show that the mini 35s-enhancer-Csy4 BiP system resulted in detectable mutations, which were absent from the mini 35s-Csy4 negative control samples (**Figures 1B, C**). Deep sequencing of PCR amplicons was used to further quantify the mutation frequency at each target site. Consistent with the CAPS result, mutation frequencies of ∼10% and ∼12% were found for* OsPDS* and *OsDEP1* target sites, respectively (**Figures 1D, E**).

### Targeted Mutagenesis in Rice T0 Lines With the Mini 35s-Enhancer BiP CRISPR-Cas9 System

**Figure 2:**
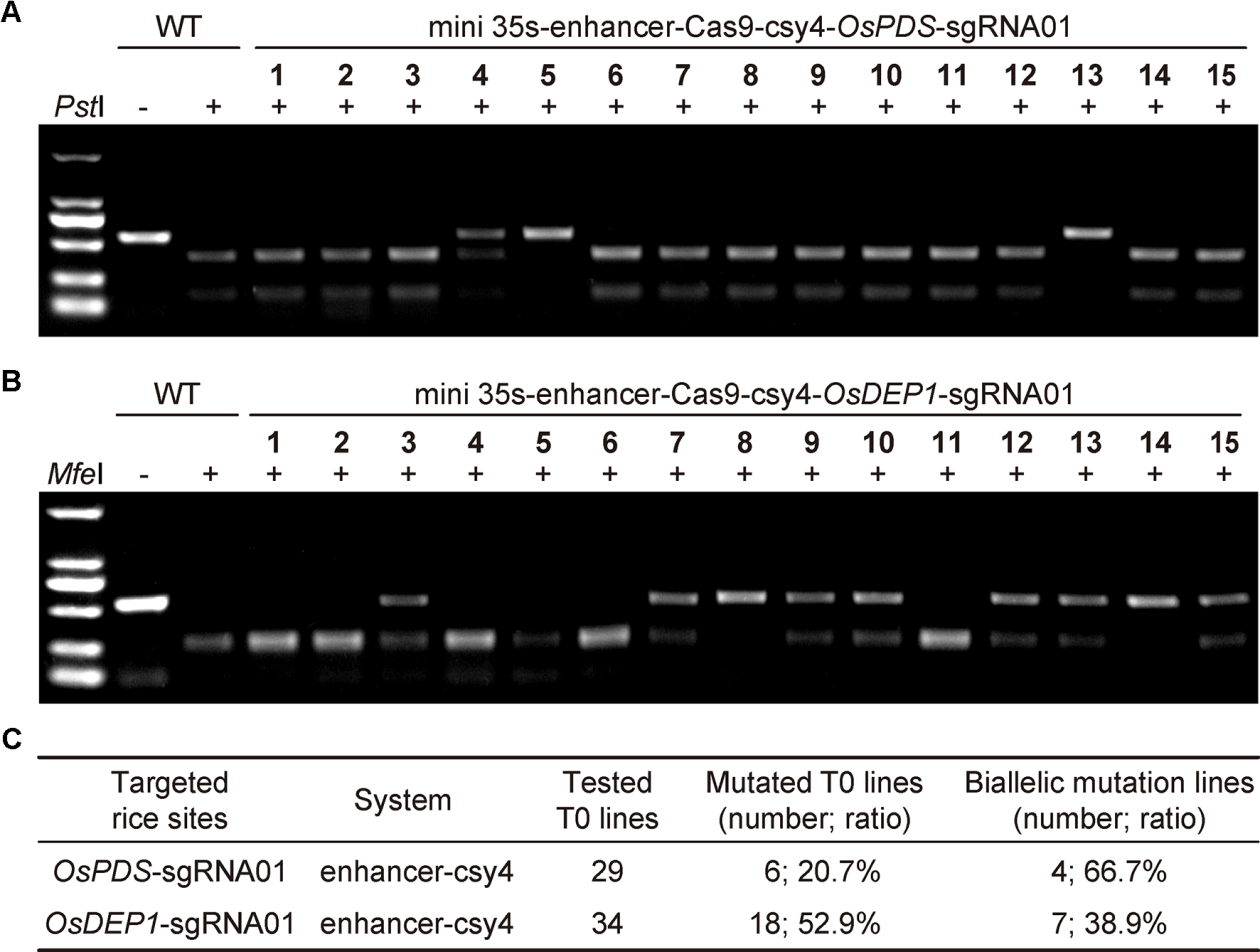
Generation of rice T0 mutants with CRISPR-Cas9 expressed by an engineered bidirectional promoter **(A)** Cleaved amplified polymorphic sequence (CAPS) analysis for targeted mutations at *OsPDS* among T0 lines. Note that only results of the first 15 lines were shown. **(B)** CAPS analysis for targeted mutations at *OsDEP1* among T0 lines. Note that only results of the first 15 lines were shown. **(C)** Summary of genotyping results of all T0 lines analyzed.

To further assess the mini 35S-enhancer-Csy4 BiP system, we used the two working T-DNA constructs to generate stable transgenic rice lines. For the *OsPDS* target site, we screened 29 T0 lines using CAPS with restriction digestion by *Pst*I (**Figure 2A**). For the *OsDEP1* target site, we screened 34 T0 lines using CAPS with restriction digestion by *Mfe*I (**Figure 2B**). Mutant lines with uncut bands were subjected for Sanger sequencing and decoding for genotype. Among 29 T0 lines in which *OsPDS* was targeted, six lines carried mutations (20.69%), in which four were biallelic and two were heterozygous (**Figure 2C** and additional data: **Figure S1**). Among 34 T0 lines in which *OsDEP1* was targeted, 18 lines carried mutations (52.94%) in which seven were biallelic and the remainder were heterozygous (**Figure 2C**). The genome editing frequencies at both target sites appeared to be lower than those obtained with the conventional mixed dual promoter CRISPR-Cas9 system (Lowder et al., 2015) or with the STU CRISPR-Cas9 system (Tang et al., 2016; Tang et al., 2019), indicating room for improvement.

### Improved Marker Gene Expression by a Rice Endogenous BiP System

**Figure 3:**
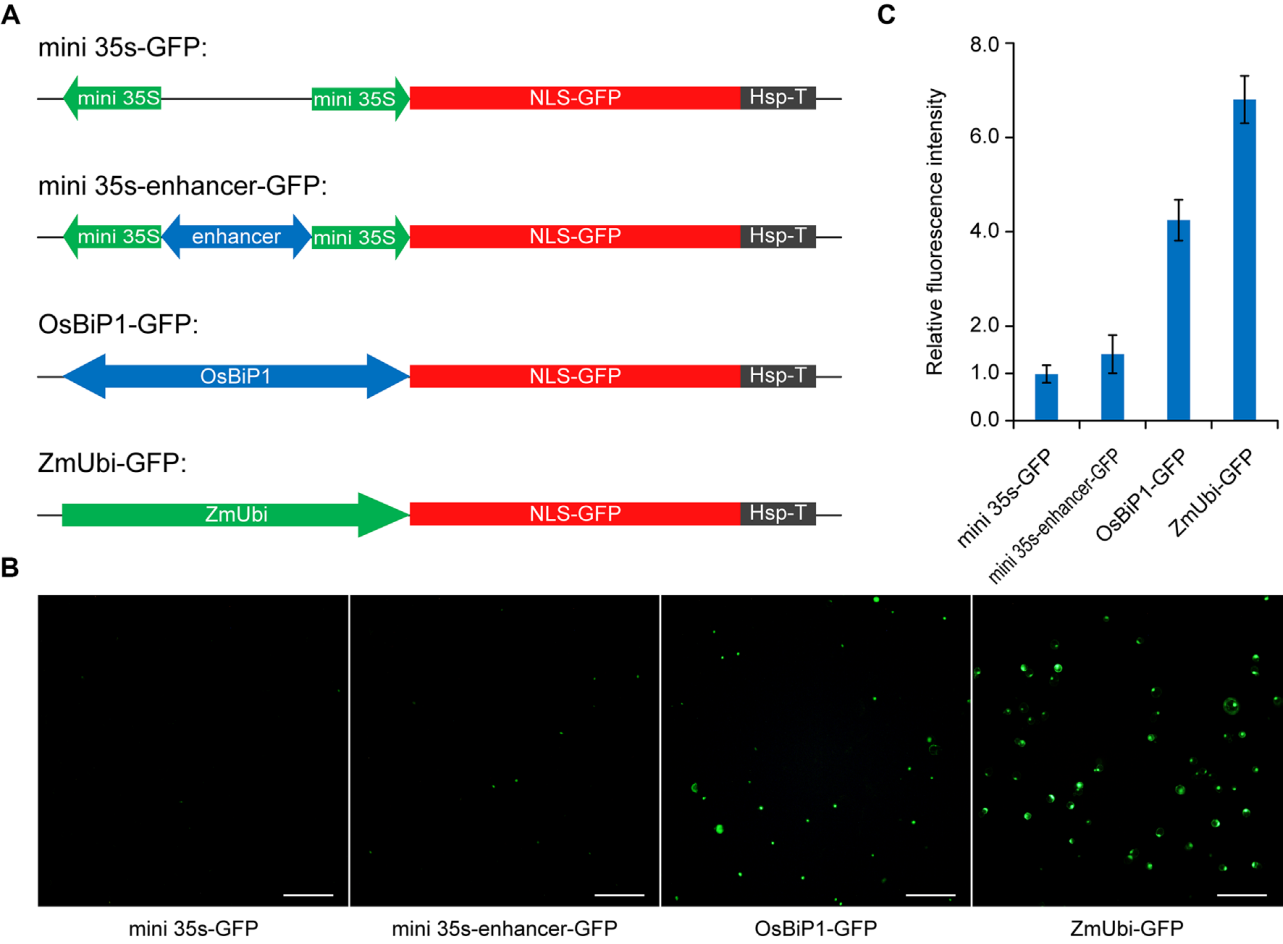
Comparison of different promoter systems in rice protoplasts **(A)** Diagrams of reporter constructs driven by different promoters. **(B)** Representative images of green-fluorescent protein (GFP)-expressing protoplasts among different treatments. **(C)** Quantification of relative fluorescence intensity for GFP-positive cells among different treatments. Bar graphs show average mutation frequency from three biologically independent replicates with error bars representing standard deviations (*n* = 3).

While we recognized that stronger BiP systems could be engineered by using stronger enhancers or multiple copies of enhancers, we reasoned that utilization of a plant endogenous BiP system may represent a straightforward strategy for improvement. Based on RNAseq and cDNA microarray data analysis, a recent study identified a constitutive BiP of high expression in rice, OsBiP1, which drives expression of Os02g42314 at the 5′ end and Os02g42320 at the 3′ end (Wang et al., 2016). We decided to test OsBiP1 in our study and compared its expression strength by positioning a GFP reporter gene at the 3′ end of this BiP. We compared OsBiP1 with the engineered mini 35s-enhancer BiP, mini 35S BiP (negative control), and ZmUbi, which is a strong unidirectional promoter commonly used for expression of *Cas* genes in rice and other monocots (Tang et al., 2017; Lee et al., 2019) (**Figure 3A**). We transiently transformed the four GFP constructs in rice protoplasts and measured GFP signals among total cells (**Figure 3B**). Based on the quantification of GFP-positive cells (**Figure 3B**) and GFP intensity in positive cells (**Figure 3C**), OsBiP1 displayed stronger expression than mini 35s-enhancer and mini 35s BiPs yet weaker expression than the ZmUbi promoter. Nevertheless, the results suggest that the use of plant endogenous BiPs may improve expression over the engineered mini 35s-enhancer system.

### The OsBip1 BiP System Results in Improved CRISPR-Cas9 Genome Editing in Rice Cells

**Figure 4:**
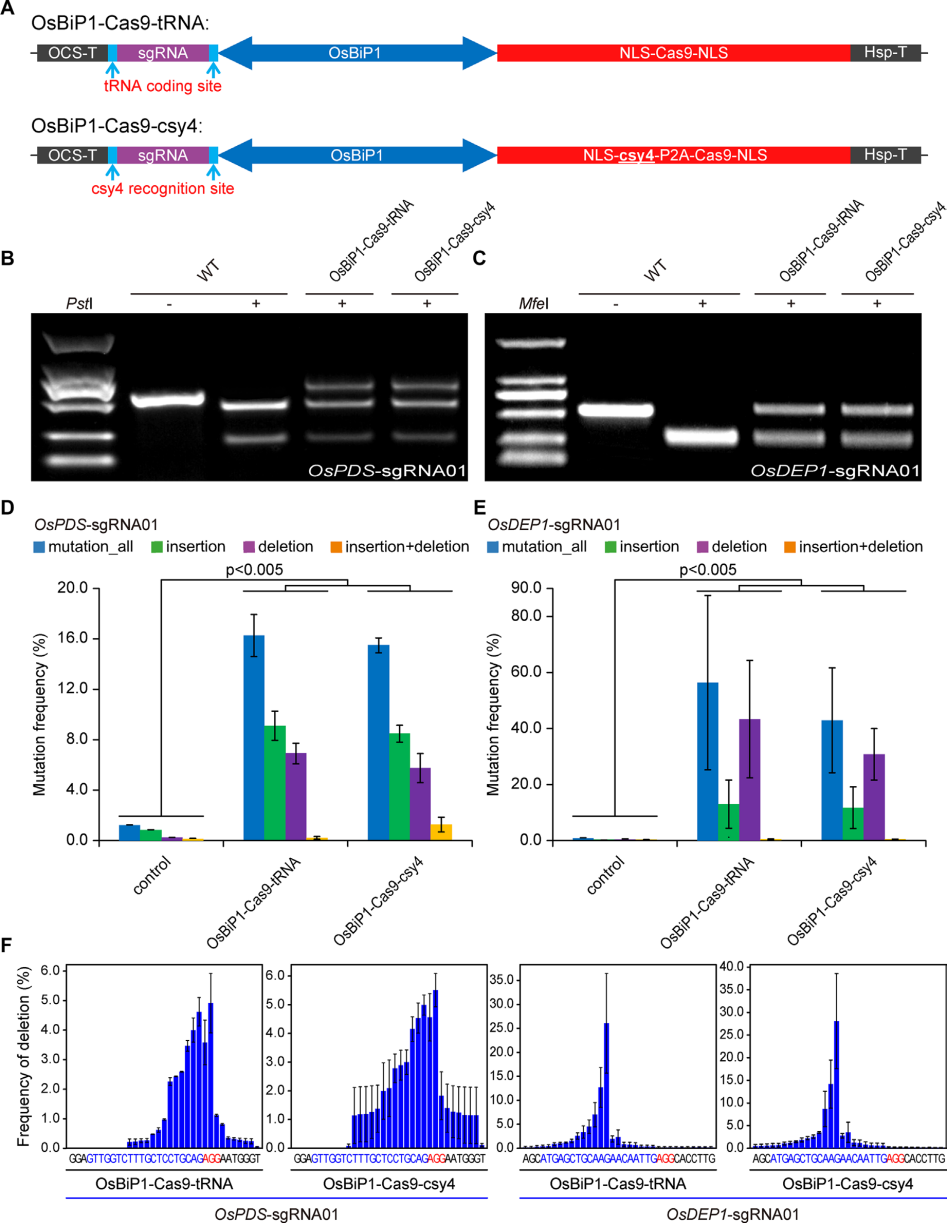
Improved editing efficiency with the OsBiP1 bidirectional promoter **(A)** Diagrams of bidirectional promoter (BiP) CRISPR-Cas9 expression constructs in which single guide RNAs (sgRNAs) are processed by tRNA and Csy4. **(B)** Cleaved amplified polymorphic sequence (CAPS) analysis of mutagenesis efficiency at *OsPDS*. **(C)** CAPS analysis of mutagenesis efficiency at *OsDEP1*. **(D)** Quantification of mutagenesis at the *OsPDS* target site by deep sequencing. **(E)** Quantification of mutagenesis at the *OsDEP1* target site by deep sequencing. **(F)** Mutation profile on deletion positions at the *OsPDS* and *OsDEP1* target sites by OsBiP1-Cas9-tRNA and OsBiP1-Cas9-Csy4. Bar graphs show average mutation frequency from three biologically independent replicates with error bars representing standard deviations (*n* = 3). The statistical analyses were performed using the two-tailed test.

We then tested OsBiP1 for the expression of CRISPR-Cas9. In our design, the sgRNA was put at the 5′ end of OsBiP1 and Cas9 was put at the 3′ end. We also sought to compare the Csy4 system with tRNA, which is another efficient polycistronic sgRNA processing system (Xie et al., 2015) (**Figure 4A**). Both OsBiP1-Cas9-tRNA and OsBiP1-Cas9-Csy4 systems were used to target the same two target sites at *OsPDS* and *OsDEP1* in rice protoplasts. In both cases, comparable mutation frequencies were detected at either target site with both systems (**Figures 4B, C**). Deep sequencing of PCR amplicons revealed mutation frequency of ∼16% at *OsPDS* and ∼40% at *OsDEP1* (**Figures 4D, E**). The mutation frequencies by OsBiP1 were significantly higher (*t*-test, *p* < 0.005) than those by the engineered mini 35s-enhancer BiP system (**Figures 1B–E**). At the *OsPDS* target site, there were more insertions than deletions (**Figure 4D**). At the *OsDEP1* target site, there were more deletions than insertions (**Figure 4E**). These data were consistent with earlier observations (**Figures 1D, E**). We then looked into deletion profiles regarding deletion positions. Consistent with mutation frequencies, the deletion profiles were largely similar for tRNA and Csy4 at the *OsPDS* target site and *OsDEP1* target site (**Figure 4F**).

### High-Efficiency Genome Editing in Rice T0 Lines With the OsBiP1 BiP CRISPR-Cas9 System

**Figure 5:**
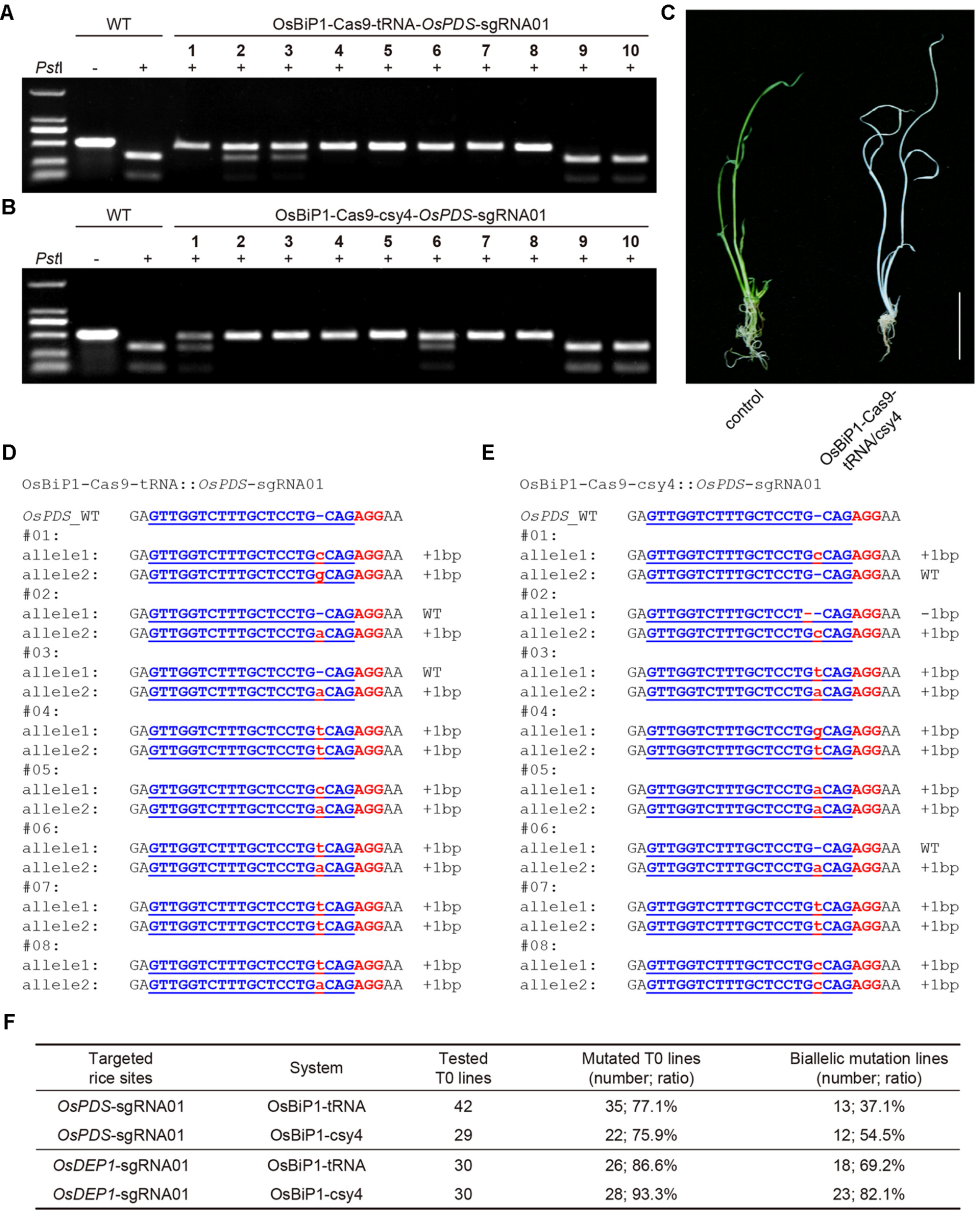
High-efficiency generation of rice T0 mutants with the OsBiP1-based CRISPR-Cas9 system **(A)** Cleaved amplified polymorphic sequence (CAPS) analysis for targeted mutagenesis at *OsPDS* among 10 T0 lines. **(B)** CAPS analysis for targeted mutagenesis at *OsDEP1* among 10 T0 lines. **(C)** A representative photo showing the wild-type control (left) and an *OsPDS* biallelic knockout plant (right). **(D)** Genotyping results of T0 lines with *OsPDS* targeted by OsBiP1-Cas9-tRNA. Note that only results from eight out of 42 T0 lines are shown. **(E)** Genotyping results of T0 lines *OsPDS* targeted by OsBiP1-Cas9-Csy4. Note that only results from 8 out of 29 T0 lines are shown. Indel events are highlighted in red in lowercase. The PAM is highlighted in red in uppercase. **(F)** Summary of genotyping results for all T0 lines targeted by OsBiP1-Cas9-tRNA and OsBiP1-Cas99-Csy4 at *OsPDS* and *OsDEP1*, respectively.

To test whether the OsBiP1 CRISPR-Cas9 system could lead to high-frequency targeted mutagenesis in stable transgenic rice lines, we generated many T0 plants for each of the four T-DNA constructs targeting *OsPDS* and *OsDEP1*. CAPS analysis revealed high frequency of uncut bands in these T0 lines with OsBiP1-Cas9-tRNA and OsBiP1-Cas9-Csy4 targeting *OsPDS* (**Figures 5A, B**), suggesting biallelic or homozygous mutations. Indeed, loss-of-function albino phenotype was observed for such lines (**Figure 5C**). The genotypes of these mutants were confirmed with Sanger sequencing. Interestingly, most T0 mutant lines carried 1-bp insertion/deletion (indel) mutations (**Figures 5D, E**). T0 lines with OsBiP1-Cas9-tRNA and OsBiP1-Cas9-Csy4 targeting *OsDEP1* were also genotyped by CAPS and Sanger sequencing (additional data: **Figures S2** and S3). At *OsPDS*, OsBiP1-Cas9-tRNA and OsBiP1-Cas9-Csy4 resulted in mutation frequencies of 77.1% and 75.9%, respectively (**Figure 5F**). Among them, biallelic mutation frequencies were 37.1% and 54.5%. At *OsDEP1*, OsBiP1-Cas9-tRNA and OsBiP1-Cas9-Csy4 generated mutation frequencies of 86.6% and 93.3%, respectively (**Figure 5F**). Among them, biallelic mutation frequencies were 69.2% and 82.1%. These results suggest significant improvement over the engineered mini 35s-enhancer BiP system. The OsBiP1-based CRISPR-Cas9 system appeared to have similar genome editing efficiency to our previously established mixed dual promoter or STU systems (Lowder et al., 2015; Tang et al., 2016; Tang et al., 2019).

## Discussion

Conventionally, CRISPR-Cas9 is expressed by a mixed dual promoter strategy in which Cas9 is expressed by a Pol II promoter and the sgRNA is expressed by a Pol III promoter such as U6 or U3. Most studies in plants have applied this mixed dual promoter systems as demonstrated in those initial studies showcasing CRISPR-Cas9 functionality in plants (Jiang et al., 2013; Li et al., 2013; Nekrasov et al., 2013; Fauser et al., 2014). Under this strategy, multiple sgRNAs can be expressed by stacking sgRNA expression cassettes (Li et al., 2013; Lowder et al., 2015), by using a tRNA processing system (Xie et al., 2015; Tang et al., 2019), by using hammerhead ribozyme (HH)–hepatitis delta virus ribozyme (HDV) (Gao and Zhao, 2014; He et al., 2017; Tang et al., 2019), or by using the Csy4 RNase system (Cermak et al., 2017; Tang et al., 2019). While it is convenient to use Pol III promoters for sgRNA expression, these promoters generate shorter transcripts and tend to have weaker expression strength than do some Pol II promoters. Additionally, the use of Pol II promoters for sgRNA expression would allow for better spatiotemporal control when needed. For coordinated expression, it is more desirable to have Cas9 and the sgRNA under the same or similar promoters. Hence, it is very valuable to develop CRISPR-Cas9 expression systems in which sgRNAs are also expressed by Pol II promoters.

There are generally three strategies to express guide RNAs by Pol II promoters. The first strategy is a STU strategy that Cas9 and sgRNA(s) are expressed by a single Pol II promoter as a single transcript. This strategy has been demonstrated for single or multiplexed genome editing in plants with sgRNAs being processed by HH ribozyme (Tang et al., 2016; Tang et al., 2019), tRNA (Tang et al., 2019), Csy4 (Tang et al., 2019), or plant endogenous RNase activities (Mikami et al., 2017). The STU strategies represent the most compact CRISPR expression systems. Given Cas and the sgRNA are expressed from the same transcript, it leaves little room for fine-tuning both components for optimal production of the Cas-guide RNA RNP complex. The second strategy is a dual Pol II promoter strategy where Cas9 and the sgRNA are expressed by separate Pol II expression units. For example, high-efficiency genome editing was achieved by using a strong Pol II promoter (CmYLCV) for expressing sgRNAs, and the editing frequencies were almost twice as much as those obtained with a Pol III promoter (AtU6) (Cermak et al., 2017). Consistent with this result, a dual Pol II promoter system also contributed to highly efficient genome editing by CRISPR-Cas12a (Tang et al., 2017; Zhong et al., 2018; Tang et al., 2019). The third strategy is a BiP system that uses a single Pol II promoter to drive Cas9 and sgRNAs in opposite directions, an idea explored in our study here. An artificial or engineered BiP system was previously demonstrated in plants (Xie et al., 2001) and was recently applied for coordinated multi-gene expression in maize for developing gene stacked GM plants (Kumar et al., 2015). It is also very common to find endogenous BiP systems in plants, such as the BiP that drives expression of *Cab1* and *Cab2* in *Arabidopsis* (Mitra et al., 2009). In this study, we showed that both engineered BiP and plant endogenous BiP systems can be used for expression CRISPR-Cas for efficient genome editing.

Among four possible CRISPR-Cas9 expression strategies (e.g., mixed dual promoters, dual Pol II promoters, STU, and BiP), a BiP system balances expression strength, compactness, and fine-tunability. In this proof-of-concept study, the rice endogenous OsBiP1 system is more efficient than the engineered BiP system based on the mini 35s promoter. We, however, want to point out that this may not be always the case because both BiP strategies, whether engineered or endogenous, can be further improved. First, rational design-based approach could be used to engineer BiP systems with improved expression strength and stability in plants, as was done in *Escherichia coli* and yeast (Yang et al., 2013; Elison et al., 2018). Second, aided by genomic and transcriptome data sets, many endogenous BiP systems could be identified and tested, as was done in rice (Wang et al., 2016). Third, different 3′-UTR and terminator sequences could be tested for tuning the expression of Cas9 and guide RNAs as well as tissue specificity (Lianoglou et al., 2013). Finally, even though we only worked with CRISPR-Cas9 in this study, it is conceivable that BiP strategies are readily applicable for the expression of CRISPR-Cas12a for genome editing and CRISPR-Cas13 for transcriptome editing (Abudayyeh et al., 2017). All these exiting fronts are awaiting future exploration.

## Data Availability

All datasets generated for this study are included in the manuscript and the Supplementary Files.

## Author Contributions

YZ conceived the project. YZ, YQ, and TZ designed the experiments. QR and XT generated all constructs. QR and ZZ performed the transient assays in protoplasts. QR, YW, QL, MY, YH, CQ, and XZ generated stable transgenic rice and identified the rice mutants. QY and TZ conducted the next-generation sequencing (NGS) data analysis. YZ, YQ, QR, and ZZ analyzed the data and wrote the manuscript draft. All authors read and approved the final manuscript.

## Conflict of Interest Statement

The authors declare that the research was conducted in the absence of any commercial or financial relationships that could be construed as a potential conflict of interest.

## Supplementary Material

Figure S1 T0 rice plants targeted by OsPDS-sgRNA01 with the mini 35s¬enhancer‐Cas9-Csy4 system. Sanger sequencing results for six T0 lines. The target sequence is shown in blue. Indel events are highlighted in red in lower case. The PAM is highlighted in red in upper case.

Figure S2 T0 rice plants targeted by OsDEP1-sgRNA01 with the OsBiP1‐Cas9-tRNA system. (A) The OsBiP1-Cas9-tRNA system indel events were first identified by CAPS analysis. (B) Sanger sequencing results for T0 lines. The target sequence is shown in blue. Indel events are highlighted in red in lower case. The PAM is highlighted in red in upper case.

Figure S3 T0 rice plants targeted by OsDEP1-sgRNA01 with the OsBiP1‐Cas9-Csy4 system. (A) The OsBiP1-Cas9-Csy4 system indel events were first identified by CAPS analysis. (B) Sanger sequencing results for T0 lines. The target sequence is shown in blue. Indel events are highlighted in red in lower case. The PAM is highlighted in red in upper case.

Figure S4 Sequence of an Arabidopsis enhancer used in this study.

Figure S5 The OsBiP1 bidirectional promoter sequence used in this study.
