# Supplementary material for: Bidirectional Promoter-Based CRISPR-Cas9 Systems for Plant Genome Editing
Source: Front Plant Sci. 2019 Sep 20;10:1173. doi: 10.3389/fpls.2019.01173 (PMC6764340; doi:10.3389/fpls.2019.01173)
Supplement: Figure S1 — T0 rice plants targeted by OsPDS-sgRNA01 with the mini 35s¬enhancer‐Cas9-Csy4 system. Sanger sequencing results for six T0 lines. The target sequence is shown in blue. Indel events are highlighted in red in lower case. The PAM is highlighted in red in upper case. [file Presentation_1.pptx]

## Slide 1
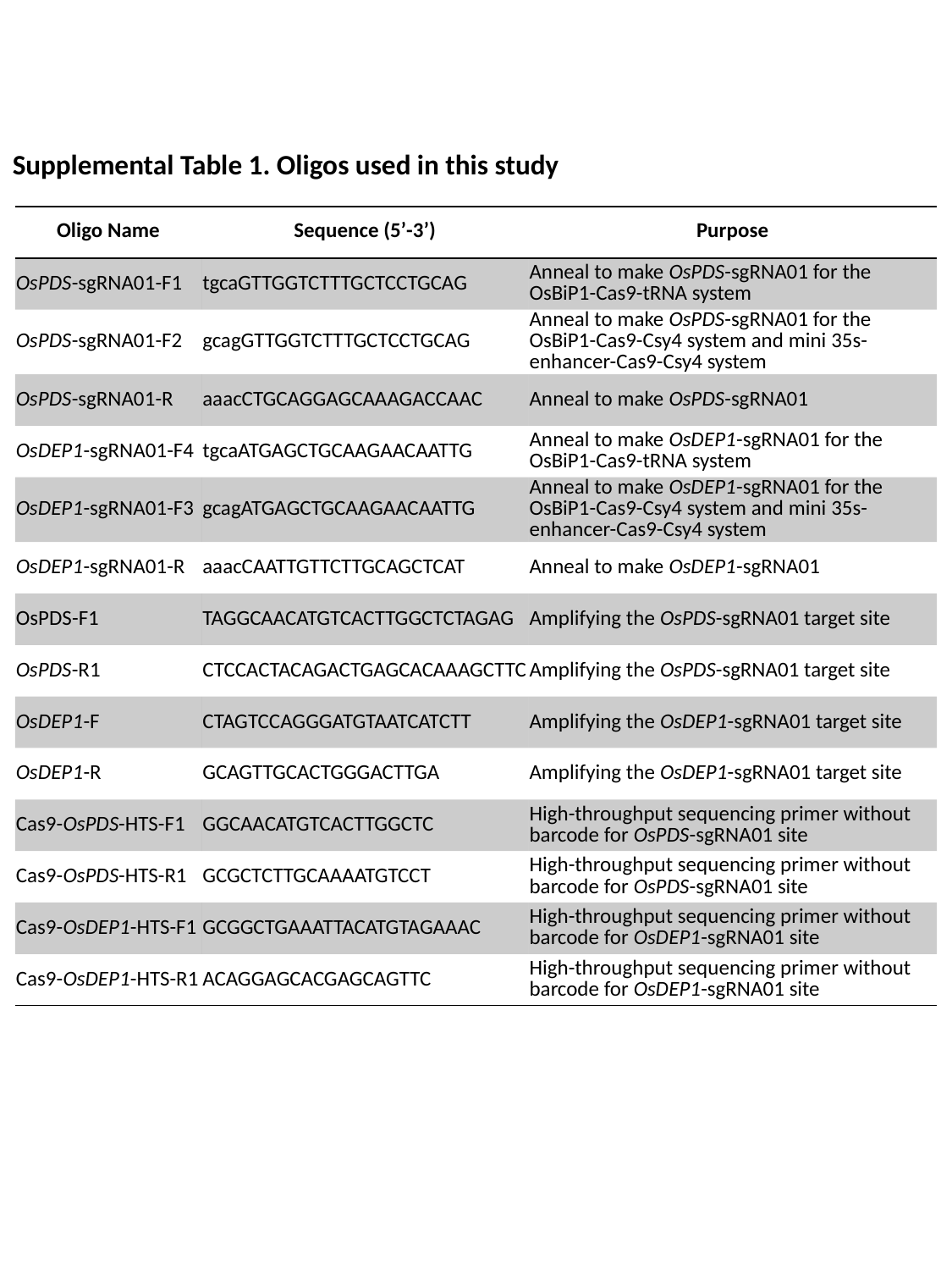

Supplemental Table 1. Oligos used in this study
| Oligo Name | Sequence (5’-3’) | Purpose |
| --- | --- | --- |
| OsPDS-sgRNA01-F1 | tgcaGTTGGTCTTTGCTCCTGCAG | Anneal to make OsPDS-sgRNA01 for the OsBiP1-Cas9-tRNA system |
| OsPDS-sgRNA01-F2 | gcagGTTGGTCTTTGCTCCTGCAG | Anneal to make OsPDS-sgRNA01 for the OsBiP1-Cas9-Csy4 system and mini 35s-enhancer-Cas9-Csy4 system |
| OsPDS-sgRNA01-R | aaacCTGCAGGAGCAAAGACCAAC | Anneal to make OsPDS-sgRNA01 |
| OsDEP1-sgRNA01-F4 | tgcaATGAGCTGCAAGAACAATTG | Anneal to make OsDEP1-sgRNA01 for the OsBiP1-Cas9-tRNA system |
| OsDEP1-sgRNA01-F3 | gcagATGAGCTGCAAGAACAATTG | Anneal to make OsDEP1-sgRNA01 for the OsBiP1-Cas9-Csy4 system and mini 35s-enhancer-Cas9-Csy4 system |
| OsDEP1-sgRNA01-R | aaacCAATTGTTCTTGCAGCTCAT | Anneal to make OsDEP1-sgRNA01 |
| OsPDS-F1 | TAGGCAACATGTCACTTGGCTCTAGAG | Amplifying the OsPDS-sgRNA01 target site |
| OsPDS-R1 | CTCCACTACAGACTGAGCACAAAGCTTC | Amplifying the OsPDS-sgRNA01 target site |
| OsDEP1-F | CTAGTCCAGGGATGTAATCATCTT | Amplifying the OsDEP1-sgRNA01 target site |
| OsDEP1-R | GCAGTTGCACTGGGACTTGA | Amplifying the OsDEP1-sgRNA01 target site |
| Cas9-OsPDS-HTS-F1 | GGCAACATGTCACTTGGCTC | High-throughput sequencing primer without barcode for OsPDS-sgRNA01 site |
| Cas9-OsPDS-HTS-R1 | GCGCTCTTGCAAAATGTCCT | High-throughput sequencing primer without barcode for OsPDS-sgRNA01 site |
| Cas9-OsDEP1-HTS-F1 | GCGGCTGAAATTACATGTAGAAAC | High-throughput sequencing primer without barcode for OsDEP1-sgRNA01 site |
| Cas9-OsDEP1-HTS-R1 | ACAGGAGCACGAGCAGTTC | High-throughput sequencing primer without barcode for OsDEP1-sgRNA01 site |

## Slide 2
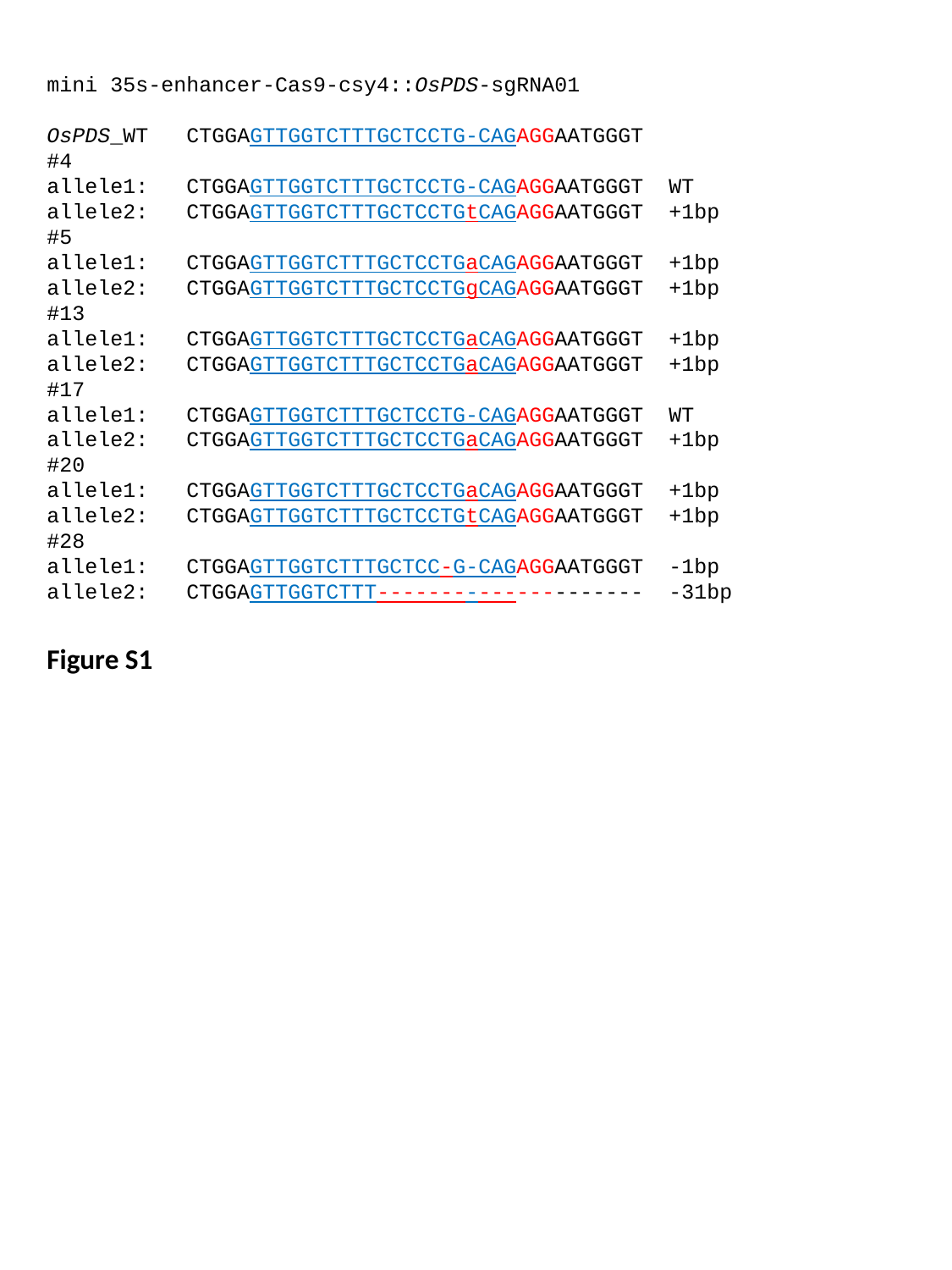

mini 35s-enhancer-Cas9-csy4::OsPDS-sgRNA01
OsPDS_WT CTGGAGTTGGTCTTTGCTCCTG-CAGAGGAATGGGT
#4
allele1: CTGGAGTTGGTCTTTGCTCCTG-CAGAGGAATGGGT WT
allele2: CTGGAGTTGGTCTTTGCTCCTGtCAGAGGAATGGGT +1bp
#5
allele1: CTGGAGTTGGTCTTTGCTCCTGaCAGAGGAATGGGT +1bp
allele2: CTGGAGTTGGTCTTTGCTCCTGgCAGAGGAATGGGT +1bp
#13
allele1: CTGGAGTTGGTCTTTGCTCCTGaCAGAGGAATGGGT +1bp
allele2: CTGGAGTTGGTCTTTGCTCCTGaCAGAGGAATGGGT +1bp
#17
allele1: CTGGAGTTGGTCTTTGCTCCTG-CAGAGGAATGGGT WT
allele2: CTGGAGTTGGTCTTTGCTCCTGaCAGAGGAATGGGT +1bp
#20
allele1: CTGGAGTTGGTCTTTGCTCCTGaCAGAGGAATGGGT +1bp
allele2: CTGGAGTTGGTCTTTGCTCCTGtCAGAGGAATGGGT +1bp
#28
allele1: CTGGAGTTGGTCTTTGCTCC-G-CAGAGGAATGGGT -1bp
allele2: CTGGAGTTGGTCTTT--------------------- -31bp
Figure S1

## Slide 3
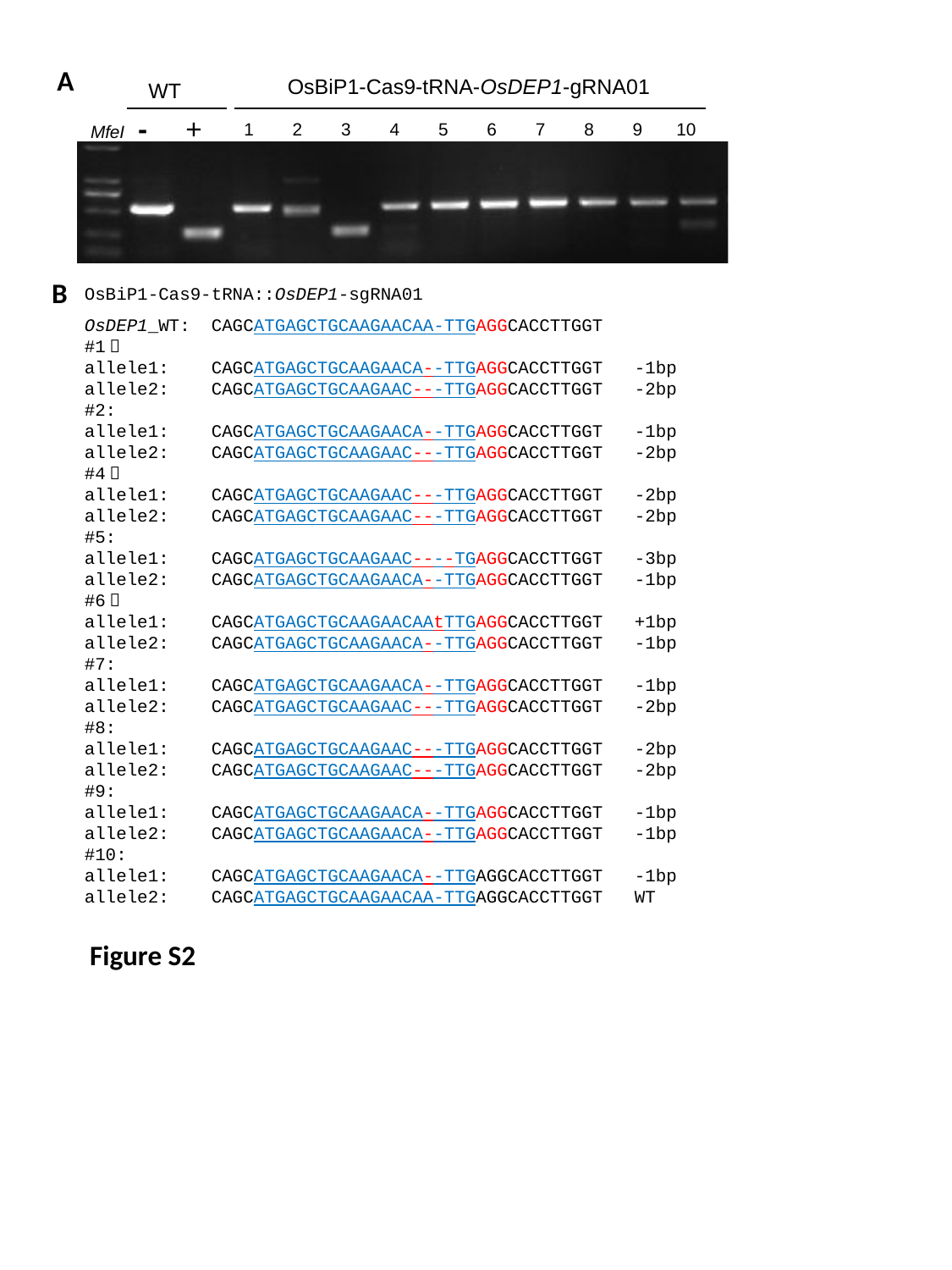

A
OsBiP1-Cas9-tRNA-OsDEP1-gRNA01
WT
-
+
 1 2 3 4 5 6 7 8 9 10
MfeI
B
OsBiP1-Cas9-tRNA::OsDEP1-sgRNA01
OsDEP1_WT: CAGCATGAGCTGCAAGAACAA-TTGAGGCACCTTGGT
#1：
allele1: CAGCATGAGCTGCAAGAACA--TTGAGGCACCTTGGT -1bp
allele2: CAGCATGAGCTGCAAGAAC---TTGAGGCACCTTGGT -2bp
#2:
allele1: CAGCATGAGCTGCAAGAACA--TTGAGGCACCTTGGT -1bp
allele2: CAGCATGAGCTGCAAGAAC---TTGAGGCACCTTGGT -2bp
#4：
allele1: CAGCATGAGCTGCAAGAAC---TTGAGGCACCTTGGT -2bp
allele2: CAGCATGAGCTGCAAGAAC---TTGAGGCACCTTGGT -2bp
#5:
allele1: CAGCATGAGCTGCAAGAAC----TGAGGCACCTTGGT -3bp
allele2: CAGCATGAGCTGCAAGAACA--TTGAGGCACCTTGGT -1bp
#6：
allele1: CAGCATGAGCTGCAAGAACAAtTTGAGGCACCTTGGT +1bp
allele2: CAGCATGAGCTGCAAGAACA--TTGAGGCACCTTGGT -1bp
#7:
allele1: CAGCATGAGCTGCAAGAACA--TTGAGGCACCTTGGT -1bp
allele2: CAGCATGAGCTGCAAGAAC---TTGAGGCACCTTGGT -2bp
#8:
allele1: CAGCATGAGCTGCAAGAAC---TTGAGGCACCTTGGT -2bp
allele2: CAGCATGAGCTGCAAGAAC---TTGAGGCACCTTGGT -2bp
#9:
allele1: CAGCATGAGCTGCAAGAACA--TTGAGGCACCTTGGT -1bp
allele2: CAGCATGAGCTGCAAGAACA--TTGAGGCACCTTGGT -1bp
#10:
allele1: CAGCATGAGCTGCAAGAACA--TTGAGGCACCTTGGT -1bp
allele2: CAGCATGAGCTGCAAGAACAA-TTGAGGCACCTTGGT WT
Figure S2

## Slide 4
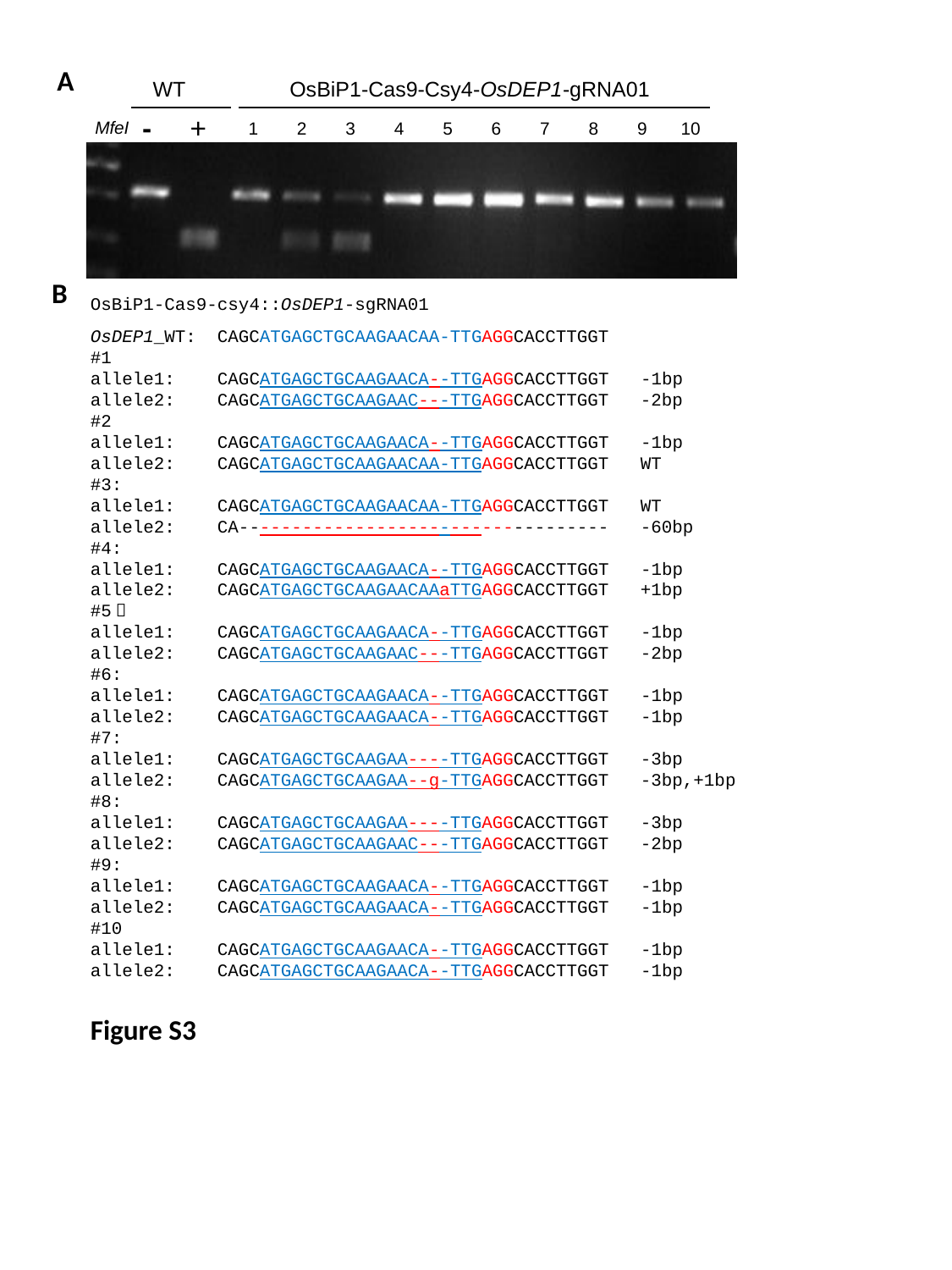

A
OsBiP1-Cas9-Csy4-OsDEP1-gRNA01
WT
-
+
MfeI
 1 2 3 4 5 6 7 8 9 10
B
OsBiP1-Cas9-csy4::OsDEP1-sgRNA01
OsDEP1_WT: CAGCATGAGCTGCAAGAACAA-TTGAGGCACCTTGGT
#1
allele1: CAGCATGAGCTGCAAGAACA--TTGAGGCACCTTGGT -1bp
allele2: CAGCATGAGCTGCAAGAAC---TTGAGGCACCTTGGT -2bp
#2
allele1: CAGCATGAGCTGCAAGAACA--TTGAGGCACCTTGGT -1bp
allele2: CAGCATGAGCTGCAAGAACAA-TTGAGGCACCTTGGT WT
#3:
allele1: CAGCATGAGCTGCAAGAACAA-TTGAGGCACCTTGGT WT
allele2: CA----------------------------------- -60bp
#4:
allele1: CAGCATGAGCTGCAAGAACA--TTGAGGCACCTTGGT -1bp
allele2: CAGCATGAGCTGCAAGAACAAaTTGAGGCACCTTGGT +1bp
#5：
allele1: CAGCATGAGCTGCAAGAACA--TTGAGGCACCTTGGT -1bp
allele2: CAGCATGAGCTGCAAGAAC---TTGAGGCACCTTGGT -2bp
#6:
allele1: CAGCATGAGCTGCAAGAACA--TTGAGGCACCTTGGT -1bp
allele2: CAGCATGAGCTGCAAGAACA--TTGAGGCACCTTGGT -1bp
#7:
allele1: CAGCATGAGCTGCAAGAA----TTGAGGCACCTTGGT -3bp
allele2: CAGCATGAGCTGCAAGAA--g-TTGAGGCACCTTGGT -3bp,+1bp
#8:
allele1: CAGCATGAGCTGCAAGAA----TTGAGGCACCTTGGT -3bp
allele2: CAGCATGAGCTGCAAGAAC---TTGAGGCACCTTGGT -2bp
#9:
allele1: CAGCATGAGCTGCAAGAACA--TTGAGGCACCTTGGT -1bp
allele2: CAGCATGAGCTGCAAGAACA--TTGAGGCACCTTGGT -1bp
#10
allele1: CAGCATGAGCTGCAAGAACA--TTGAGGCACCTTGGT -1bp
allele2: CAGCATGAGCTGCAAGAACA--TTGAGGCACCTTGGT -1bp
Figure S3

## Slide 5
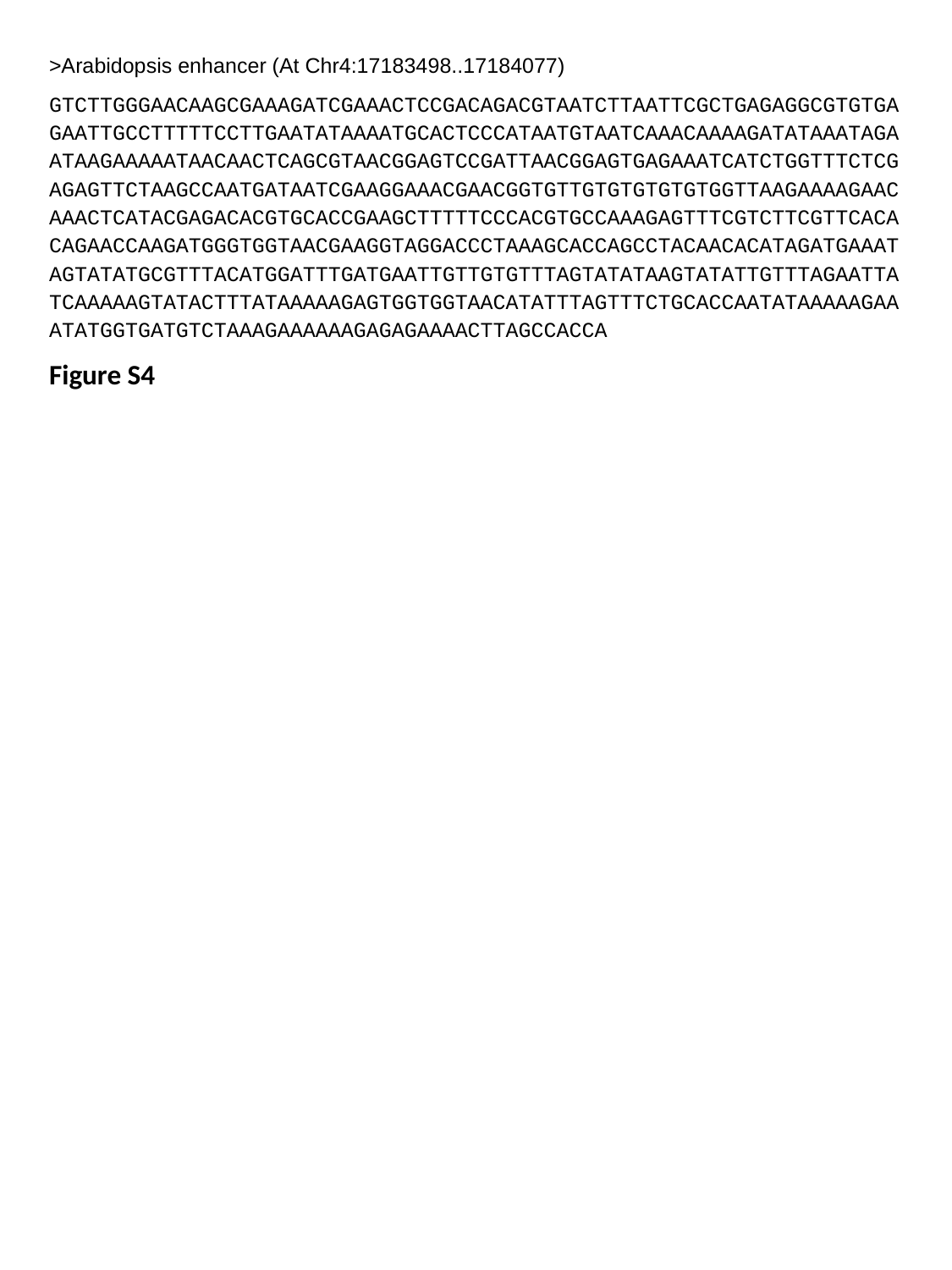

>Arabidopsis enhancer (At Chr4:17183498..17184077)
GTCTTGGGAACAAGCGAAAGATCGAAACTCCGACAGACGTAATCTTAATTCGCTGAGAGGCGTGTGAGAATTGCCTTTTTCCTTGAATATAAAATGCACTCCCATAATGTAATCAAACAAAAGATATAAATAGAATAAGAAAAATAACAACTCAGCGTAACGGAGTCCGATTAACGGAGTGAGAAATCATCTGGTTTCTCGAGAGTTCTAAGCCAATGATAATCGAAGGAAACGAACGGTGTTGTGTGTGTGTGGTTAAGAAAAGAACAAACTCATACGAGACACGTGCACCGAAGCTTTTTCCCACGTGCCAAAGAGTTTCGTCTTCGTTCACACAGAACCAAGATGGGTGGTAACGAAGGTAGGACCCTAAAGCACCAGCCTACAACACATAGATGAAATAGTATATGCGTTTACATGGATTTGATGAATTGTTGTGTTTAGTATATAAGTATATTGTTTAGAATTATCAAAAAGTATACTTTATAAAAAGAGTGGTGGTAACATATTTAGTTTCTGCACCAATATAAAAAGAAATATGGTGATGTCTAAAGAAAAAAGAGAGAAAACTTAGCCACCA
Figure S4

## Slide 6
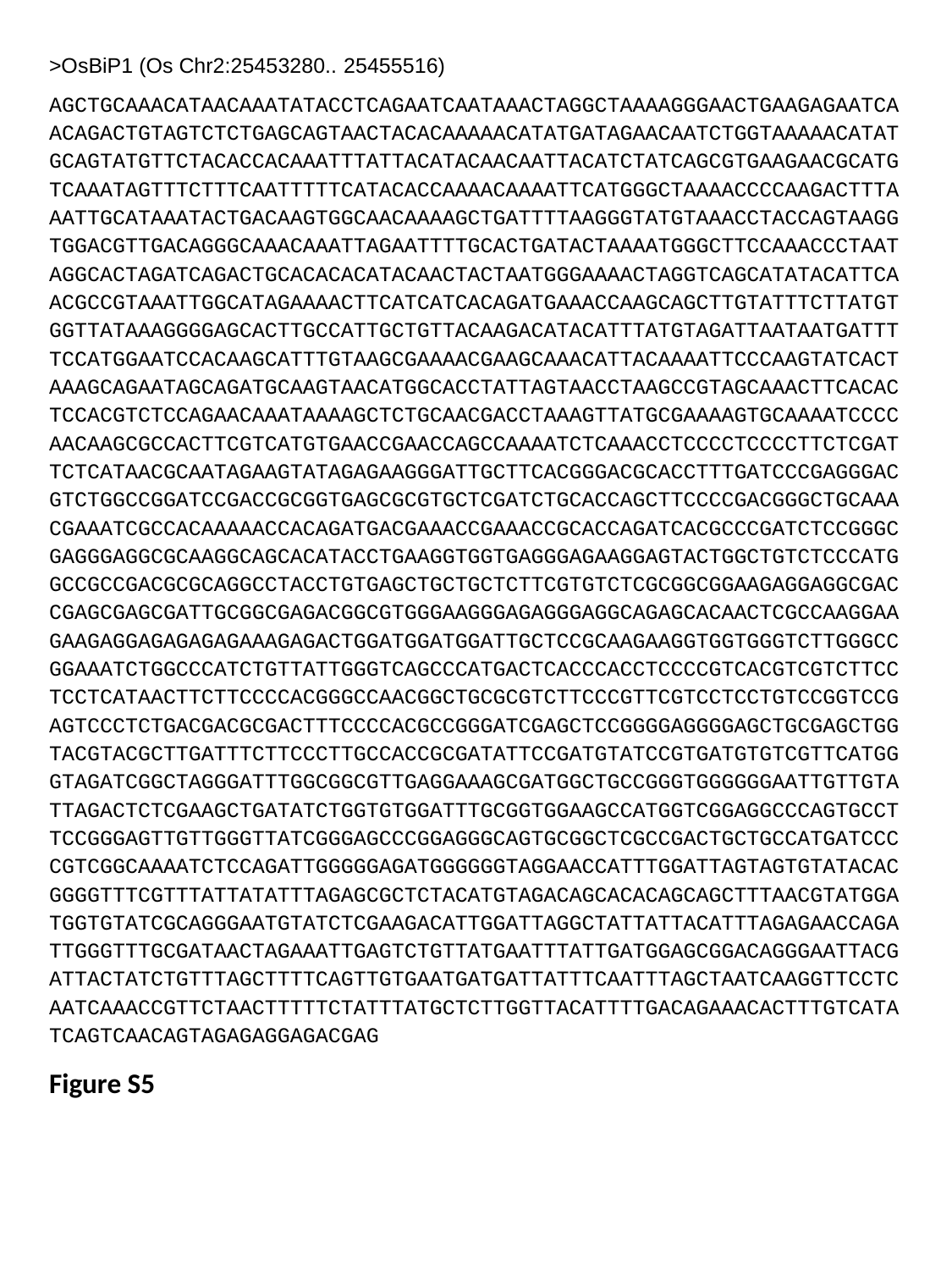

>OsBiP1 (Os Chr2:25453280.. 25455516)
AGCTGCAAACATAACAAATATACCTCAGAATCAATAAACTAGGCTAAAAGGGAACTGAAGAGAATCAACAGACTGTAGTCTCTGAGCAGTAACTACACAAAAACATATGATAGAACAATCTGGTAAAAACATATGCAGTATGTTCTACACCACAAATTTATTACATACAACAATTACATCTATCAGCGTGAAGAACGCATGTCAAATAGTTTCTTTCAATTTTTCATACACCAAAACAAAATTCATGGGCTAAAACCCCAAGACTTTAAATTGCATAAATACTGACAAGTGGCAACAAAAGCTGATTTTAAGGGTATGTAAACCTACCAGTAAGGTGGACGTTGACAGGGCAAACAAATTAGAATTTTGCACTGATACTAAAATGGGCTTCCAAACCCTAATAGGCACTAGATCAGACTGCACACACATACAACTACTAATGGGAAAACTAGGTCAGCATATACATTCAACGCCGTAAATTGGCATAGAAAACTTCATCATCACAGATGAAACCAAGCAGCTTGTATTTCTTATGTGGTTATAAAGGGGAGCACTTGCCATTGCTGTTACAAGACATACATTTATGTAGATTAATAATGATTTTCCATGGAATCCACAAGCATTTGTAAGCGAAAACGAAGCAAACATTACAAAATTCCCAAGTATCACTAAAGCAGAATAGCAGATGCAAGTAACATGGCACCTATTAGTAACCTAAGCCGTAGCAAACTTCACACTCCACGTCTCCAGAACAAATAAAAGCTCTGCAACGACCTAAAGTTATGCGAAAAGTGCAAAATCCCCAACAAGCGCCACTTCGTCATGTGAACCGAACCAGCCAAAATCTCAAACCTCCCCTCCCCTTCTCGATTCTCATAACGCAATAGAAGTATAGAGAAGGGATTGCTTCACGGGACGCACCTTTGATCCCGAGGGACGTCTGGCCGGATCCGACCGCGGTGAGCGCGTGCTCGATCTGCACCAGCTTCCCCGACGGGCTGCAAACGAAATCGCCACAAAAACCACAGATGACGAAACCGAAACCGCACCAGATCACGCCCGATCTCCGGGCGAGGGAGGCGCAAGGCAGCACATACCTGAAGGTGGTGAGGGAGAAGGAGTACTGGCTGTCTCCCATGGCCGCCGACGCGCAGGCCTACCTGTGAGCTGCTGCTCTTCGTGTCTCGCGGCGGAAGAGGAGGCGACCGAGCGAGCGATTGCGGCGAGACGGCGTGGGAAGGGAGAGGGAGGCAGAGCACAACTCGCCAAGGAAGAAGAGGAGAGAGAGAAAGAGACTGGATGGATGGATTGCTCCGCAAGAAGGTGGTGGGTCTTGGGCCGGAAATCTGGCCCATCTGTTATTGGGTCAGCCCATGACTCACCCACCTCCCCGTCACGTCGTCTTCCTCCTCATAACTTCTTCCCCACGGGCCAACGGCTGCGCGTCTTCCCGTTCGTCCTCCTGTCCGGTCCGAGTCCCTCTGACGACGCGACTTTCCCCACGCCGGGATCGAGCTCCGGGGAGGGGAGCTGCGAGCTGGTACGTACGCTTGATTTCTTCCCTTGCCACCGCGATATTCCGATGTATCCGTGATGTGTCGTTCATGGGTAGATCGGCTAGGGATTTGGCGGCGTTGAGGAAAGCGATGGCTGCCGGGTGGGGGGAATTGTTGTATTAGACTCTCGAAGCTGATATCTGGTGTGGATTTGCGGTGGAAGCCATGGTCGGAGGCCCAGTGCCTTCCGGGAGTTGTTGGGTTATCGGGAGCCCGGAGGGCAGTGCGGCTCGCCGACTGCTGCCATGATCCCCGTCGGCAAAATCTCCAGATTGGGGGAGATGGGGGGTAGGAACCATTTGGATTAGTAGTGTATACACGGGGTTTCGTTTATTATATTTAGAGCGCTCTACATGTAGACAGCACACAGCAGCTTTAACGTATGGATGGTGTATCGCAGGGAATGTATCTCGAAGACATTGGATTAGGCTATTATTACATTTAGAGAACCAGATTGGGTTTGCGATAACTAGAAATTGAGTCTGTTATGAATTTATTGATGGAGCGGACAGGGAATTACGATTACTATCTGTTTAGCTTTTCAGTTGTGAATGATGATTATTTCAATTTAGCTAATCAAGGTTCCTCAATCAAACCGTTCTAACTTTTTCTATTTATGCTCTTGGTTACATTTTGACAGAAACACTTTGTCATATCAGTCAACAGTAGAGAGGAGACGAG
Figure S5
